# Balancing Osmotic Protection and Oxidative Stress: Physiological and Biochemical Responses of Pot Marigold (*Calendula officinalis* L.) Plants to Water Stress

**DOI:** 10.3390/plants14243809

**Published:** 2025-12-14

**Authors:** Diana Ribeiro, Maria Rita Guzmán, Ana D. Caperta, Isabel Marques

**Affiliations:** 1 Linking Landscape, Environment, Agriculture and Food (LEAF) Research Center, Associate Laboratory TERRA, School of Agriculture (ISA), University of Lisbon, Tapada da Ajuda, 1349-017 Lisbon, Portugal; dcribeiroo20@gmail.com; 2 Forest Research Center (CEF), Associate Laboratory TERRA, School of Agriculture (ISA), University of Lisbon, Tapada da Ajuda, 1349-017 Lisbon, Portugal; 3 Estación de Biodiversidad La Ceiba, Chisec 16013, Guatemala; mrguzman@gmail.com

**Keywords:** *Calendula officinalis*, drought stress, oxidative stress, antioxidants, ornamental plants

## Abstract

Water deficit is a widespread environmental constraint that disrupts plant metabolism, impairs growth, and compromises ornamental value. In this study, we examined the integrated morpho-physiological and biochemical responses of *Calendula officinalis* L. to moderate (MWS; 60% field capacity) and severe (SWS; 35% field capacity) drought, compared with well-watered plants, over a three-week period under controlled conditions. Drought stress triggered pronounced reductions in vegetative growth: leaf number decreased by 33.1% under MWS and 51.0% under SWS, and leaf length declined by 34.7% and 42.7%, respectively. Fresh and dry biomass decreased significantly, especially under SWS where it was accompanied by a decrease in leaf water loss capacity. Non-enzymatic antioxidant responses included a decline in carotenoid content and strong osmolyte accumulation, with proline increasing under SWS. Indicators of oxidative damage, hydrogen peroxide (H_2_O_2_) and malondialdehyde (MDA) also rose, but only under SWS. In parallel, the enzymatic antioxidant system (catalase, peroxidase and superoxide dismutase) was significantly activated under drought. Our results demonstrate that *C. officinalis* uses a dual drought response, combining osmotic adjustment with an upregulation of antioxidant defenses to limit oxidative stress. However, under prolonged severe drought, these mechanisms are insufficient to prevent biomass loss, underscoring its vulnerability in water-limited environments.

## 1. Introduction

Water deficit, which occurs when evapotranspiration exceeds water input over time, is one of the most common abiotic stresses experienced by plants. Under the current scenarios of global climate change, prolonged periods of reduced rainfall and increasing evaporative demand have intensified the frequency and severity of droughts, threatening food and ornamental plant production, most especially across arid and semi-arid regions [1]. According to recent estimates, drought episodes and heatwaves have tripled in frequency over the last five decades and now affect over 40% of the global agricultural area [2,3]. Consequently, understanding how plants perceive and respond to water limitation is not only of scientific interest but also of strategic importance for sustainable agriculture.

Water deficit disrupts nearly every level of plant organization, from root water uptake to leaf gas exchange and reproductive development, ultimately constraining productivity and quality [4]. Physiologically, a common early reaction is the closure of stomata, usually mediated by abscisic acid (ABA), which reduces water loss but concurrently limits CO_2_ uptake and photosynthetic efficiency [5,6]. Cell expansion also diminishes due to a reduction in turgor pressure, and a decline in nutrient uptake since water mobility in the rhizosphere also decreases, further limiting plant growth and morphogenesis. As a result, drought-stressed plants frequently show a reduction in leaf number and size, in order to minimize transpiration surface area [7,8]. These morphological adjustments lead to changes in plant architecture, often accompanied by visible symptoms of dehydration, such as leaf wilting, rolling, or chlorosis [9], especially under prolonged or severe stress [10].

Plant responses to drought involve integrated adjustments in water relations, metabolism, and gene regulation. The perception of osmotic stress in root and shoot tissues triggers calcium-dependent signaling cascades and the activation of transcription factors such as *DREB*, *NAC*, and *WRKY* families, which regulate downstream protective genes [11]. Hormonal crosstalk plays a central role, with ABA, jasmonic acid, and salicylic acid pathways, coordinating growth inhibition with defense activation and protection against stress [12]. Osmotic adjustment is another key physiological mechanism. Plants accumulate compatible solutes such as soluble sugars (sucrose, trehalose), amino acids (proline, glycine betaine), and polyols (mannitol, sorbitol), which maintain cell turgor and protect macromolecules from denaturation [13]. These osmolytes also stabilize proteins and membranes, preserving enzyme activity and photosynthetic capacity under reduced water potential. Moreover, membrane stability becomes critical during dehydration [4]. Inside cells, the imbalance between light harvesting and electron transport under limited CO_2_ assimilation promotes the excessive formation of reactive oxygen species (ROS) such as superoxide radicals (O_2_•^−^) and hydrogen peroxide (H_2_O_2_) [14]. While low concentrations of ROS function as signaling molecules involved in stress acclimation, their overaccumulation damages proteins, lipids, and nucleic acids. The resulting oxidative stress can lead to membrane rupture, enzyme inhibition, and accelerated senescence if detoxification mechanisms fail [14].

To cope with oxidative stress, plants rely on a coordinated antioxidant defense network. Enzymatic components, including superoxide dismutase (SOD), catalase (CAT), ascorbate peroxidase (APX), and peroxidase (POX), are usually activated to stabilize ROS levels [15]. Superoxide dismutase (SOD) catalyzes the dismutation of superoxide radicals (O_2_•^−^) into hydrogen peroxide (H_2_O_2_), which is subsequently broken down by CAT or APX into water and oxygen. Ascorbate peroxidase (POX) contributes to H_2_O_2_ removal using phenolic compounds as electron donors, maintaining cellular redox homeostasis and protecting against oxidative damage [16,17]. Non-enzymatic antioxidants, such as carotenoids, flavonoids, and phenolic acids, further complement enzymatic defenses by scavenging singlet oxygen and hydroxyl radicals [18,19]. These compounds also act as signaling molecules that regulate stress-responsive genes and maintain redox signaling pathways. Osmoprotectants also contribute to drought tolerance not only by osmotic adjustment but also through multiple regulatory roles [13]. Proline, for instance, acts as a molecular chaperone stabilizing enzymes and membranes, and buffers cytosolic pH under stress. It also contributes with electrons to the mitochondrial respiration and functions as a redox modulator through proline dehydrogenase-mediated oxidation, being notable for its multifunctional role in many different crops such as maize [20], roses [21], or pot marigolds (*Calendula* sp.) [22]. Proline aids not only in stress protection [23], but also in preserving tissue turgor and structural integrity under water-limited conditions [24,25]. This metabolic flexibility enables plants to maintain energy balance and protect against dehydration-induced cytotoxicity. Malondialdehyde (MDA), a by-product of lipid peroxidation, serves as a reliable biochemical marker for oxidative damage and is frequently used to quantify the severity of drought-induced stress [26]. High MDA accumulation indicates compromised membrane integrity, whereas lower levels under drought reflect an efficient antioxidant response. Thus, together, these biochemical parameters provide a comprehensive framework for evaluating plant tolerance to water stress.

*Calendula officinalis* L., a fast growing annual belonging to the Asteraceae family, has a substantial economic and ecological value due to its use in cosmetics, pharmaceuticals, and herbal supplements [27]. These plants produce composite flower heads (inflorescences) with yellow-orange ligulate florets rich in carotenoids, which are valued for therapeutic treatments and as a cost-effective culinary substitute for saffron [28,29]. Traditionally known as the ‘sun herb’ due to the tendency of flowers to open at sunrise and close at sunset, the species has been used in herbal medicine since the 12th century [27]. While it is native to Central Europe, the Mediterranean region, and the Middle East, it is currently widely cultivated in countries such as India, China, and the United States [27]. The species contributes to agroecosystem biodiversity by attracting pollinators and natural enemies of pests [30]. Phytochemically, the plant is rich in triterpenoids (faradiol esters), flavonoids, carotenoids, and essential oils that confer anti-inflammatory, antimicrobial, and antioxidant properties [30]. Notwithstanding these applications, few works have studied genetic diversity or physiological responses in marigold plants [31,32].

The global market for *C. officinalis* flower extracts was estimated at USD 200–250 million in 2022, with projections suggesting a growth to USD 400 million by around 2030–2033, at a compound annual growth rate of roughly 6–8% [33]. In 2023, the extract market alone was valued at USD 85–150 million, with cosmetics and skincare accounting for around 50% of total demand, followed by pharmaceutical applications (~30%). The fastest-growing segments include tinctures and medicinal preparations, especially in North America, Europe, and Asia-Pacific. Additionally, estimates indicate that the broader pot marigold extract market, encompassing oils and powders, reached nearly USD 350 million in 2024, with oils alone contributing about USD 210 million [34]. This economic footprint highlights *C. officinalis* as an important species with significant market presence across multiple industries, reinforcing its relevance to sustainable floriculture and multichannel value chains.

Despite these advantages, drought affects the growth and productivity of pot marigold plants. A significant reduction in both fresh and dry biomass has been consistently reported in *C. officinalis* under drought conditions [22,35,36]. Drought generates yield reductions ranging from 4–7% under moderate water stress (e.g., 75% field capacity) to 24–40% under severe stress (e.g., 50% field capacity) but strongly depending on the genotype/cultivar used [22]. For instance, in the ‘Sun Glow’ cultivar, flower production losses of up to 39.9% were documented under severe drought [22]. Thus, while these figures may vary between genotypes, they clearly illustrate the substantial vulnerability of *C. officinalis* cultivation to water limitations. Given the agronomic and economic importance of pot marigold plants, improving their drought resilience is critical for maintaining yield and product quality under changing environmental conditions. Therefore, this study aimed to understand the morphological, osmotic, and antioxidant defense responses of *C. officinalis* to progressive drought stress, providing mechanistic insights into the coordination between growth regulation and oxidative stress mitigation. These findings help to support future efforts in selecting resilient cultivars, optimizing irrigation practices, and applying targeted agronomic interventions to sustain plant quality and yield under water-limited conditions.

## 2. Results

### 2.1. Effects of Drought on Vegetative Structures

Water limitation had a pronounced effect on several vegetative traits of pot marigolds (Table 1). The number of leaves decreased significantly under water deficit (F_2,12_ = 6.307, *p* = 0.013). Plants exposed to both moderate water stress (MWS) and severe water stress (SWS) produced fewer leaves than well-watered controls. On average, plants under MWS exhibited a 33.1% reduction (−5.2 leaves) and those under SWS a 51.0% reduction (−8.0 leaves) compared to control plants. Drought stress also impacted leaf length, with control plants exhibiting longer leaves compared to drought-stressed ones, although the difference was only marginally significant (F_2,12_ = 3.796, *p* = 0.048). This corresponds to a 34.7% and 42.7% reduction, respectively. In contrast, leaf width did not differ significantly among treatments (F_2,12_ = 3.453, *p* = 0.065), representing reductions of approximately 38.2% (MWS) and 37.3% (SWS), respectively.

Fresh and dry biomass measurements confirmed the detrimental impact of water deficit on vegetative growth. The fresh weight (FW) of leaves declined from 171.0 ± 18.0 mg in control plants to 150.0 ± 12.0 mg in plants under MWS (−12.1%) and 62.0 ± 5.0 mg under SWS (−63.6%) (F_2,12_ = 21.024, *p* < 0.001). Dry weight (DW) followed a similar pattern, dropping from 13.1 ± 1.0 mg in control leaves to 9.0 ± 2.0 mg under MWS (−32.2%) and 6.0 ± 1.0 mg under SWS (−53.9%) (F_2,12_ = 8.282, *p* < 0.001). Leaf water loss calculations indicated a sharp decline in water retention capacity, dropping from 0.676 ± 0.76 in control leaves, decreasing to −0.695 ± 2.34 under MWS (−202.8%), and to −11.687 ± 1.81 under SWS. The latter reflects a substantial disruption of water balance and tissue turgor under severe drought.

### 2.2. Activities of Non-Enzymatic and Stress-Related Metabolites

**Figure 1:**
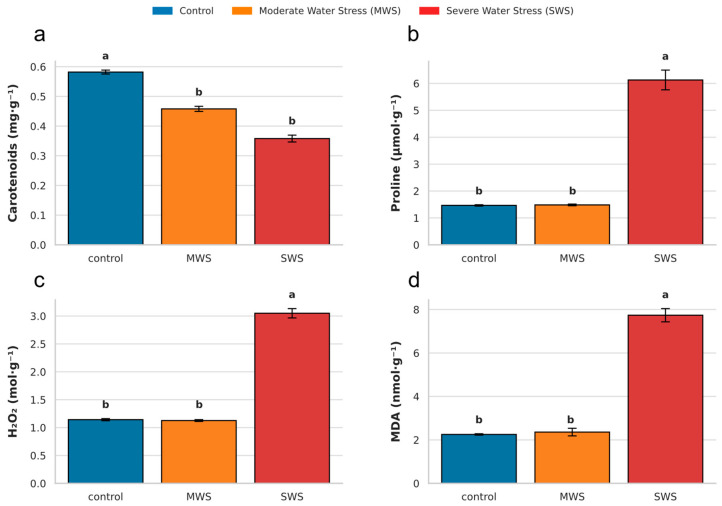
Mean levels of antioxidant-related metabolites in pot marigold (*Calendula officinalis*) leaves under different water treatments: control (blue); moderate water stress, MWS (orange), and severe water stress, SWS (red). Metabolites measured include (**a**) carotenoids (mg·g^−1^), (**b**) proline (µmol·g^−1^), (**c**) hydrogen peroxide—H_2_O_2_ (mol·g^−1^), and (**d**) malondialdehyde—MDA (nmol·g^−1^). Bars represent mean values ± standard error. Different letters above bars indicate statistically significant differences among treatments for each metabolite (ANOVA, Tukey HSD post hoc, *p* < 0.05).

Water availability significantly influenced the levels of several antioxidant-related metabolites in pot marigolds (Figure 1). Carotenoid content decreased significantly under water stress (F_2,12_ = 149,905, *p* < 0.001), with reductions of approximately 15.5% under MWS and 19.0% under SWS, compared to control plants (Figure 1a). In contrast, proline levels increased significantly, but only under the harsh drought treatment (F_2,12_ = 159.025, *p* < 0.001), where it rose by 34.7% (Figure 1b). The accumulation of this amino acid reflects osmotic adjustment and protective responses against dehydration. A similar trend was observed for hydrogen peroxide (H_2_O_2_), which increased significantly but only under SWS (8.3%; F_2,12_ = 488.848, *p* < 0.001) (Figure 1c). Malondialdehyde (MDA) content, an indicator of lipid peroxidation and oxidative membrane damage, also increased significantly under SWS (F_2,12_ = 238.233, *p* < 0.001), with levels raised by 8.9% compared with control plants (Figure 1d). Together, these results indicate that while moderate drought induced minimal biochemical disturbance, severe stress disrupted redox homeostasis, leading to pigment loss, oxidative damage, and accumulation of osmolytes.

### 2.3. Enzymatic Activities in Response to Drought

**Figure 2:**
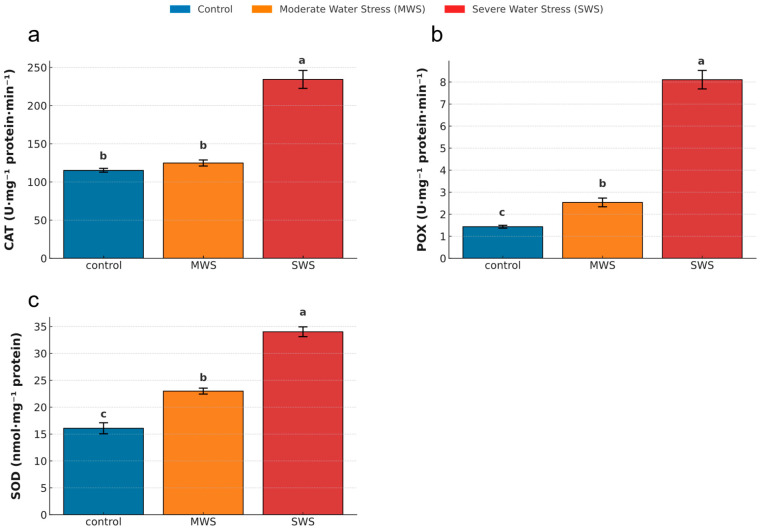
Mean enzymatic antioxidant activities in leaves of *Calendula officinalis* under different water treatments: control (blue); moderate water stress, MWS (orange), and severe water stress, SWS (red). Enzymes include (**a**) catalase (CAT), (**b**) peroxidase (POX), and (**c**) superoxide dismutase (SOD). Bars represent mean values ± standard error. Different letters above bars indicate statistically significant differences among treatments for each enzyme (ANOVA, Tukey HSD post hoc test, *p* < 0.05).

All analyzed antioxidant enzymes exhibited enhanced activity under drought stress (Figure 2), though the magnitude of response varied among enzymes and treatments. Catalase (CAT) activity rose under water stress, although only significantly under the harsher stress (F_2,12_ = 93.709, *p* < 0.01). A 129.5% increase was recorded in SWS plants compared to control ones (Figure 2a). Peroxidase (POX) activity increased even more sharply, showing a 76.9% rise under MWS and a 465.4% increase under SWS relative to control conditions (Figure 2b; F_2,12_ = 175.345, *p* < 0.01). Superoxide dismutase (SOD) activity also increased progressively under water deficit (F_2,12_ = 112.719, *p* < 0.01). Results show an increase of 43.0% under MWS and 111.6% under SWS compared to control conditions (Figure 2c). These coordinated enzymatic adjustments demonstrate that *C. officinalis* enhances its antioxidant machinery in response to declining water availability, with maximal up-regulation under severe stress.

**Figure 3:**
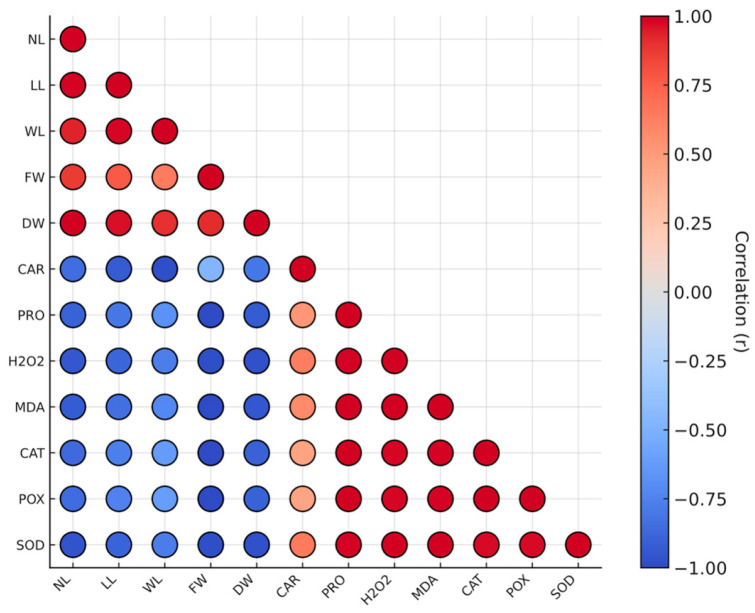
Correlation matrix among morphological, osmotic, and antioxidant traits of *Calendula officinalis* plants subjected to water stress. Color intensity represent the strength and direction of correlation (red = positive, blue = negative; *p* < 0.05). Figure acronyms indicate the number of leaves (NL), length of leaves (LL), width of leaves (WL), fresh weight (FW), dry weight (DW), carotenoids (CAR), proline (PRO), hydrogen peroxide (H_2_O_2_) malondialdehyde (MDA), catalase (CAT), peroxidase (POX), and superoxide dismutase (SOD).

Correlation analysis revealed clear and consistent relationships among growth, osmotic, and oxidative variables (Figure 3). A strong negative association was observed between biomass-related traits (leaf number, fresh and dry weight) and oxidative stress markers (MDA and H_2_O_2_). Similarly, proline showed a strong positive correlation with the activities of antioxidant enzymes (r = 0.78–0.86, *p* < 0.01), confirming that osmotic adjustment is closely coupled with enzymatic defense activation.

Oxidative parameters (MDA, H_2_O_2_) were also positively correlated with antioxidant enzymes (r = 0.82–0.91, *p* < 0.01), suggesting that ROS accumulation acts as a signal triggering antioxidant activity rather than merely reflecting cell damage. Carotenoid content was moderately correlated with biomass traits (r ≈ 0.65–0.70, *p* < 0.05) and negatively correlated with MDA (r ≈ –0.68), highlighting its protective role in maintaining membrane stability and photosynthetic pigments under stress.

## 3. Discussion

Drought is one of the most pervasive abiotic stresses affecting plant growth and productivity worldwide, and it triggers a range of morphological, physiological, and biochemical responses to cope with water-limited conditions. In this study, we demonstrate that water deficit significantly affected *Calendula officinalis* plants, reducing the number of leaves, their size and biomass, with responses depending on the severity of the drought. The decline in the number of leaves (−33% under moderate water stress, MWS and −51% under severe water stress, SWS) and their length (−35% and −43%, respectively) is consistent with a drought-induced inhibition of cell expansion caused by a decrease in turgor pressure and in photosynthetic assimilation of carbon [37], particularly under severe drought. Similar results have been reported in other ornamental crops. For instance, drought reduced leaf thickness, palisade tissue, and specific leaf area in seven different varieties of lilies, despite enhanced antioxidant and osmotic adjustments [38]. Comparable growth suppression was observed in *Rosa damascena* that showed a marked decrease in shoot and total plant biomass when grown under water-deficit conditions, together with a significant decline in chlorophyll content and water potential [39]. Drought also decreased shoot biomass, leaf area, net photosynthesis, chlorophyll *a* fluorescence, and relative water content in *Callistemon citrinus* and *Viburnum tinus* [40]. Thus, these findings confirm that drought commonly induces morphological changes that can impact both growth and ornamental value. The stress imposed by drought often triggers an excessive accumulation of ROS, resulting in oxidative damage to lipids, proteins, and nucleic acids [17,41]. In this study, we infer the presence of oxidative stress in *C. officinalis* due to the significant increase in the levels of H_2_O_2_ and MDA, especially SWS (+8% and +9%, respectively). Likewise, drought was reported to elevate H_2_O_2_ and MDA levels in *Rosa damascena* and *R. canina* [42]. In contrast, in our study, carotenoid content decreased by approximately 15.5% under MWS and 19.0% under SWS. Carotenoids act as vital precursors of phytohormones, functioning as important regulators of plant development and overall stress resilience [43,44]. Thus, our results contrast with other studies that reported an elevated carotenoid accumulation as part of a photoprotective strategy. For instance, an increase in carotenoid levels was reported in broccoli seedlings grown under drought stress [45]. Here, the observed reduction suggests either a suppression of carotenoid biosynthesis or enhanced degradation under water deficit. Consequently, this response could weaken both protective pigmentation and stress-induced hormonal signaling, leaving plants more vulnerable to drought. To overcome the harsh effects of drought, an osmotic adjustment must occur.

*Calendula officinalis* showed a significant increase in proline content, especially under SWS (+35%). Proline, a compatible solute, stabilizes proteins and membranes, reduces osmotic potential, and scavenges ROS [25]. The reduction in leaf water loss observed in drought-stressed plants supports the role of osmolyte accumulation in sustaining water status under adverse conditions and suggests that proline accumulation not only contributes to cellular protection but also indirectly supports drought avoidance through structural regulation. The reduction in leaf number and area observed under stress likely minimizes transpirational losses, while osmolyte accumulation helps maintain turgor pressure in remaining tissues. This mechanism is consistent with findings reported in *Zinnia elegans* [46], where an accumulation of proline was also recorded in response to stress, and linked to tolerance mechanisms [46]. Such parallel results also suggest the potential of proline as a physiological marker for screening drought-resilient *C. officinalis* cultivars.

The strong induction of antioxidant enzymes in *C. officinalis* also supports the view that biochemical defenses play a central role in stress responses. Catalase (CAT), which increased by 129% under SWS, is a tetrameric heme-containing enzyme localized primarily in peroxisomes, promoting a rapid decomposition of hydrogen peroxide (H_2_O_2_) into water and molecular oxygen. This activity is crucial during drought because photorespiration and β-oxidation of fatty acids intensify under stomatal closure, both generating excess H_2_O_2_ in peroxisomes. By preventing H_2_O_2_ accumulation in *C. officinalis*, CAT safeguards macromolecules such as lipids and nucleic acids from oxidative damage, as also reported in other plant species [47,48]. Peroxidases (POX), a large family of heme-containing enzymes distributed across the cytosol, vacuole, and apoplast, exhibited an even greater induction in this study, rising by 77% under moderate and by 465% under severe drought stress. Unlike CAT, POX enzymes operate at lower H_2_O_2_ concentrations and use a variety of electron donors [15,49]. This versatility allows POX to participate not only in H_2_O_2_ detoxification but also in the oxidative cross-linking of cell-wall polymers and lignin biosynthesis [50], reinforcing mechanical strength and reducing water permeability. The massive increase in POX activity observed under severe stress supports this dual function. Superoxide dismutase (SOD), often considered the “frontline” antioxidant, showed up to a 112% increase in activity, in this study. SOD catalyzes the dismutation of superoxide anions (O_2_^−^•), highly reactive radicals generated in chloroplasts, mitochondria, and plasma membrane NADPH oxidases, into H_2_O_2_ and O_2_ [51]. The H_2_O_2_ generated by SOD is subsequently processed by CAT and POX, linking these enzymes into a coordinated cascade [15]. Thus, the integration of SOD, CAT, and POX activities constitutes a multilayered antioxidant network in *C. officinalis*, where ROS levels are sequentially regulated in cells.

The correlation analysis further supports a coordination between growth inhibition and biochemical defense mechanisms. Leaf number, fresh weight, and dry weight were strongly and negatively correlated with H_2_O_2_, MDA, and antioxidant enzyme activities, confirming that growth suppression is closely associated with oxidative imbalance. Conversely, positive associations among proline, CAT, POX, and SOD activities indicate a functional coupling between osmotic regulation and enzymatic ROS detoxification. These findings demonstrate that the osmoprotective and antioxidant pathways operate in tandem to buffer drought-induced oxidative stress, although this integration becomes insufficient under SWS, as reflected by the residual accumulation of ROS and lipid peroxidation markers. From another perspective, petal senescence in *C. officinalis* ‘Resina’ was accompanied by decreased SOD, CAT, and APX activities, increased lipoxygenase (LOX) and specific protease activity (SPA) [52]. At the molecular level, ABA biosynthetic gene *AAO3* was upregulated, whereas cytokinin biosynthetic gene *IPT3* and *DAD1* declined during senescence, suggesting that hormonal regulation may also complement biochemical defenses in this species [52]. The coordination of enzymatic and non-enzymatic components to minimize oxidative damage while allowing controlled ROS signaling transduction cascades was also reported in other species from the Asteraceae family. For example, H_2_O_2_, proline, and antioxidative enzyme activities increased in milk thistle plants (*Silybum marianum*) grown under drought stress despite a reduction in the leaf chlorophyll [53]. The activity of antioxidant enzymes also increased on *Stevia rebaudiana* plants grown under drought together with a decrease in plant height [54]. Nevertheless, the activity of antioxidant enzymes decreased under long drought stress exposure in *Atractylodes lancea*, together with the down-regulation of most of the genes encoding such enzymes [55], emphasizing the importance of stress duration in plant responses.

The intensity of the stress also affects the response of *C. officinalis* since, although antioxidant systems were activated, they were also insufficient to completely prevent ROS accumulation under the harsher stress, as reported above. Consistent with these results, a previous study in other pot marigold genotypes (‘Indian Prince’, ‘Golden Emperor’, ‘Orange Prince’, and ‘Sun Glow’) also found some resilience to moderate but not to severe drought [22]. These genotypes were grown under the same levels of drought and also showed an increase in the activities of antioxidant enzyme activities, proline accumulation, and MDA levels upon stress [22], indicating that these might be general responses of *C. officinalis* plants to drought stress. Increased MDA and leaf and root proline were also reported in nine other pot marigold varieties (‘Ahwaz’, ‘Tehran’, ‘Isfahan-m1’, ‘Isfahan1’, ‘Candy-man’, ‘Gitana’, ‘Zen-gold’, ‘Isfahan-m2’, and ‘Isfahan2’) grown under 35, 60, and 85% “exhaustion of available water in the soil” [56]. Despite this, as well as the high CAT, POX, and POX activities, it was not enough to prevent a reduction in seed oil content, dry petal yield, and extract yield, seed yield and seed oil yield [56], or even flower production [22]. The harsh impacts of drought in marigold plants were also reported in a recent study where plants (variety not stated) were grown in a greenhouse and subjected, at the onset of blooming (as in our study) to three irrigation levels: 100% (control), 60% (moderate stress), and 30% (severe stress) of FC [57]. Results based on restricted maximum likelihood (REML) analysis using best linear unbiased estimates (BLUEs) showed that the moderate stress (60% FC) allowed plants to maintain normal growth, with most morpho-physiological traits, including the number of flowers, flower weight and diameter, and plant height, not differing significantly from control plants [57]. In contrast, severe drought (30% FC) reduced plant height, flower diameter, and flower weight by 31%, 20%, and 38%, respectively [57]. The study also indicated that pot marigold plants respond to drought through both avoidance and tolerance strategies, such as root system elongation, modulation of antioxidant enzyme activity, and adjustments in chlorophyll and carotenoid levels. As reported here, CAT and MDA levels were elevated only in severe drought stress treatment, while the levels of carotenoids decreased with the increase in stress severity [57]. Overall, these studies also reveal that stress impacts varied widely between *C. officinalis* genotypes. The variability observed among cultivars highlights a genetic basis for drought tolerance in *C. officinalis*, likely linked to differences in antioxidant gene expression and osmolyte metabolism, that should be further tested.

Thus, the overall picture is that *C. officinalis* employs a dual strategy under drought: morphological avoidance (reducing transpiring surface) and biochemical tolerance (osmolyte accumulation and antioxidant activation). Together, these mechanisms confer partial resilience to moderate drought but are insufficient under severe stress, where growth suppression, oxidative injury, and biomass losses become evident. Maintaining water availability above 60% field capacity appears essential to preserve leaf area and biomass, which directly determine market value in ornamental crops. However, biochemical indicators such as proline, MDA, and antioxidant enzyme activities may serve as early biomarkers of stress before visible wilting or color loss occurs, and integrating such physiological markers into irrigation scheduling and precision fertigation systems could improve water-use efficiency and reduce yield losses. Moreover, given that *C. officinalis* is widely used for medicinal and cosmetic purposes, maintaining carotenoid content is crucial for product quality.

Although our results reveal important physiological and biochemical mechanisms, they do not extend to the molecular level. Future studies integrating transcriptomic or proteomic approaches could reveal the regulatory networks underlying osmolyte accumulation, ROS detoxification, and stress tolerance. Such molecular insights would complement our findings and facilitate the selection or engineering of drought-resilient cultivars. Recent evidence also suggests that nano-silicon and chelated zinc applications improve drought and salinity resilience in *C. officinalis* by regulating hormonal balance and antioxidant responses [35,58,59]. Finally, the interplay among multiple stresses is often complex. Stress interactions may be synergistic (greater than additive), additive, or antagonistic (less than additive), depending on the type, timing, and intensity of each stress. For instance, shading can mitigate the effects of stress during waterlogging at flowering by cooling the canopy [60]. In this context, future studies combining abiotic stresses such as drought and heat will allow evaluation of whether synergistic oxidative damage exceeds that observed under either stress alone and whether the drought-responsive biochemical markers identified here exhibit altered sensitivity under combined stress conditions. Similar approaches or considerations, such as optimizing light exposure [61] or irrigation timing [56], could potentially help *C. officinalis* better cope with combined stresses, supporting growth and flowering under variable environmental conditions.

## 4. Materials and Methods

### 4.1. Plant Growth and Water Treatments

In this study, seeds of *C. officinalis* L. cv. Geisha Girl were first surface sterilized with 0.1% sodium hypochlorite for 5 min, thoroughly rinsed with distilled water, and germinated in grown in 2 L capacity pots with 50% peat, 25% perlite, and 25% vermiculite following [62]. We selected this variety because it represents a distinct phylogenetic lineage within pot marigold, differing markedly from the commonly cultivated varieties [31]. It is also presumed to exhibit enhanced tolerance to drought, an aspect that, despite frequently suggested in horticultural sources, has not yet been formally evaluated, contrary to other varieties [22]. This provided a strong rationale for using this variety as a representative model to assess physiological responses under controlled water-deficit conditions. Plants were cultivated in a nursery under controlled environmental conditions (temperature 20.5–25.3 °C; relative humidity 72–76%, photoperiod 16 h light/8 h dark) for three weeks to ensure plant uniformity at the stage of two-true leaves. During this period, irrigation was performed twice weekly (50 mL per pot) using Hoagland nutritive solution containing Na_2_Fe·EDTA (20 mg L^−1^), H_3_BO_3_ (2.86 mg L^−1^), MnSO_4_·4H_2_O (2.13 mg L^−1^), ZnSO_4_·7H_2_O (0.22 mg L^−1^), CuSO_4_·7H_2_O (0.08 mg L^−1^), and (NH_4_)_6_Mo_7_O_24_ (0.02 mg L^−1^).

Seedlings were then transplanted to individual pots, containing the same substrate composition and transferred to a controlled-environment growth chamber (RUMED, Carnaxide, Portugal). with the following conditions: Growth conditions were set to a long-day photoperiod (16 h light), temperature of 23 and 19 °C during the light and dark periods, respectively, and relative humidity maintained at 72–76%. Light intensity was 700–800 μmol m^−2^ s^−1^. Each drained pot contained only one plant and was supported by a polyethylene tray to avoid cross-leaching among treatments. Plants were irrigated twice weekly with 200 mL of water and allowed to acclimatize before the imposition of stress.

At the onset of flowering (three-month-old plants), drought stress was imposed for three weeks using three irrigation regimes corresponding to different percentages of field capacity (FC): control (100% FC), moderate water stress (60% FC: MWS), and severe water stress (35% FC: SWS). These moisture levels were selected because, based on previous studies on pot marigold plants, they reflect distinct physiological thresholds where *C. officinalis* transitions from normal growth (≈100% FC) to moderate stress (≈60% FC) and to severe stress (≈35% FC) [22]. Each treatment included five biological replicates. Water deficit levels were defined as percentages of field capacity (FC) for the substrate mix. For this, FC was first determined gravimetrically by saturating the substrate, allowing drainage for 24 h, and weighing each pot to obtain the maximum water content. Desired water levels were maintained by weighing pots before irrigation and adding the required volume to restore the defined % FC. This gravimetric control was verified twice weekly. Each treatment included five independent biological replicates (n = 5).

At the end of the stress period, plant height (measured from soil level to the apex of the tallest inflorescence), maximum leaf length, and leaf width were recorded for each plant using a digital caliper. Single leaves from each replicate were weighed to determine fresh weight (FW) immediately after harvest and subsequently oven-dried at 70 °C for 72 h to determine dry weight (DW). Leaf water content and relative water loss were determined using the formula leaf water loss (g/g) = (FW − WW)/DW, where WW is the leaf weight after the defined wilting period (72 h) at room temperature (22 °C). These parameters were used to estimate the extent of dehydration and the capacity for water retention, which are critical indicators of drought tolerance in ornamental species.

### 4.2. Activities of Non-Enzymatic and Stress-Related Metabolites

To assess the biochemical responses related to osmotic adjustment and oxidative stress, representative leaves and flowers from each replicate were analyzed for carotenoids, proline, and malondialdehyde (MDA) content. The total carotenoid content was based on 500 mg of fresh flowers, which were extracted with 30 mL of 80% ice-cold acetone before being vortexed and centrifuged at 10,000× *g* for 10 min. The supernatant was separated, and its absorbance was measured at 480 nm following [63]. Results were converted and expressed as mg of carotenoids per gram of fresh weight (FW). Proline was determined according to acid–ninhydrin and toluene methods [64] and absorbance was determined at 520 nm. Results were expressed in micrograms of proline per gram of FW. Lipid peroxidation was quantified according to [65] and measured in terms of malondialdehyde content (MDA), and absorbance was measured at 532 nm. Results are expressed as nmol of MDA per gram of FW. Each assay was run in triplicate to minimize analytical variability.

### 4.3. Antioxidative Enzyme Activities

To assess maximal enzyme activity levels, 500 mg of fresh vegetative tissue (FW) was utilized. These samples were pooled from three leaves per plant and treatment. Tissues were homogenized in 1 mL of an extraction buffer composed of 200 mM Tris-HCl (pH 8.0), 10 mM MgCl_2_·6H_2_O, 30 mM β-mercaptoethanol, 4 mM DTT, 2% Triton X-100, two tablets of complete EDTA-free protease inhibitor cocktail (Roche, Basel, Switzerland), and 10% glycerol. Additionally, 1% polyvinylpolypyrrolidone was included during homogenization. The homogenates were centrifuged at 13,000× *g* for 20 min at 4 °C, and the resulting supernatants were collected for enzyme activity assays.

The activity of enzymes was then measured as described in [22]. Briefly, catalase (CAT) activity was evaluated in a 1.5 mL reaction mixture comprising 50 mM phosphate buffer (pH 7.0), 10 mM hydrogen peroxide (H_2_O_2_), and enzyme extract. The decomposition rate of H_2_O_2_ was monitored at 240 nm, and activity was reported as CAT units per milligram of protein FW, where one unit corresponds to the breakdown of 1 mM H_2_O_2_ per min. Peroxidase (POX) activity was measured by recording the change in absorbance at 430 nm. The extinction coefficient used for calculations was 2.47 mM^−1^ cm^−1^. One POX unit was defined as the enzyme quantity required to degrade 1 μmol of H_2_O_2_ per min. Results were expressed in units per mg protein FW. Ascorbate peroxidase (APX) activity was determined based on reaction mixtures that included 50 mM phosphate buffer (pH 7.8), 20 mM ascorbate, 0.1 mM H_2_O_2_, and 10 μL of enzyme extract in a final volume of 1 mL. The reaction progress was followed by the decline in absorbance at 290 nm due to ascorbate oxidation. An extinction coefficient of 2.8 mM^−1^ cm^−1^ was used, and results were expressed as APX units per mg protein DW.

### 4.4. Statistical Analyses

Data were analyzed using one-way analysis of variance (ANOVA) followed by Tukey’s HSD post hoc test (*p* < 0.05). Normality and homogeneity of variances were verified before analysis. Correlation coefficients were calculated among all measured variables to identify interdependence among growth, osmotic, and oxidative stress markers. All statistical analyses were conducted using R studio (v4.3.2).

## 5. Conclusions

The integrative assessment of morphological, physiological, and biochemical responses of *Calendula officinalis* under controlled drought conditions revealed a dynamic interplay between water status, osmotic adjustment, and oxidative stress mitigation. Moderate drought triggered adaptive mechanisms such as reduced leaf area, accumulation of osmoprotectants (proline), and enhanced activities of antioxidant enzymes (CAT, POX, and APX), which collectively minimized oxidative injury and sustained plant function. However, under severe water stress, these defense responses were insufficient to prevent oxidative cellular damage, leading to higher ROS accumulation, lipid peroxidation, and significant reductions in biomass.

Maintaining irrigation at or above 60% FC is therefore critical to sustaining the ornamental and commercial quality of *C. officinalis* while ensuring efficient water use. Among the measured traits, proline, MDA, and H_2_O_2_ contents emerged as early biochemical markers of drought stress intensity, offering practical diagnostic tools for rapid screening of drought-resilient cultivars. The pronounced activation of antioxidant enzymes under moderate stress also indicates a threshold beyond which protective mechanisms collapse, highlighting the importance of early drought management.

These findings contribute to a mechanistic understanding of how *C. officinalis* coordinates morphological and biochemical defenses under water limitation. Identifying robust physiological indicators provides a foundation for developing precision irrigation strategies and predictive models of plant performance under fluctuating climatic conditions, key steps toward sustainable ornamental horticulture in a changing environment.

## Figures and Tables

**Table 1:** Variations in the number, length (cm) and width (cm) of pot marigold (*Calendula officinalis*) leaves from plants grown under the different water treatments: control, moderate water stress (MWS) and severe water stress (SWS). Results are expressed as means ± Standard Error. Numbers between brackets indicate minimum and maximum values. Different superscript letters indicate significant differences between water levels based on ANOVA followed by a Tukey test at *p* < 0.05. Figures on the right depict the study material: (**A**) *Calendula officinalis* L. inflorescence; (**B**) Detail of the compositive flower structure; (**C**) Effects of the different water deficit treatments (control, MWS, and SWS) on the leaves of *C. officinalis*.

**Treatments**	**Number**	**Length**	**Width**	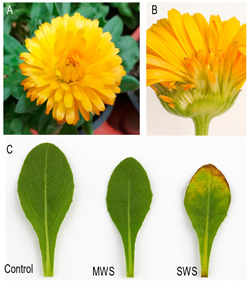
Control	15.7 ± 3.8 ^a^ (11.7–20.0)	7.5 ± 0.9 ^a^ (6.0–8.3)	2.41 ± 0.5 ^a^ (1.8–3.0)	
MWS	10.5 ± 4.2 ^a,b^ (4.0–15.0)	4.9 ± 2.8 ^b^ (0.9–6.8)	1.49 ± 0.91 ^a^ (0.24–2.31)	
SWS	7.7 ± 2.6 ^b^ (4.3–10.7)	4.3 ± 1.9 ^b^ (1.8–7.1)	1.51 ± 0.59 ^a^ (0.91–2.42)	

## Data Availability

Data is contained within the article.
